# Analyses of *Monascus* pigment secretion and cellular morphology in non‐ionic surfactant micelle aqueous solution

**DOI:** 10.1111/1751-7915.13038

**Published:** 2017-12-14

**Authors:** Gong Chen, Meihua Wang, Xiaofei Tian, Zhenqiang Wu

**Affiliations:** ^1^ School of Bioscience and Bioengineering Guangdong Provincial Key Laboratory of Fermentation and Enzyme Engineering South China University of Technology Guangzhou 510006 China; ^2^ Dongguan Tianyi Biotechnology Co. Ltd. Dongguan 523000 China

## Abstract

*Monascus* pigments produced by *Monascus spp*. are widely used as natural food colourants. Extractive fermentation technology can facilitate the secretion of intracellular *Monascus* pigments into extracellular non‐ionic surfactant micelle aqueous solution, so as to avoid the feedback inhibition and decomposition. In this study, behaviour of the trans‐membrane secretion of *Monascus* pigments was investigated using morphological and spectroscopic analyses. Laser scanning confocal microscopy (LSCM) traced that pigment secretion occurred through rapid trans‐membrane permeation in 4 min, with a simultaneous conversion in pigment characteristics. Approximately 50% of intracellular pigments (AU_470_) extracted to extracellular broth with 40 g l^−1^ Triton X‐100, indicating the capacity for pigment extraction was limited by the saturation concentrations of surfactant. Scanning electron microscope (SEM) and transmission electron microscope (TEM) imaging showed some damage in the cell wall but an intact cell membrane with a slightly increased mycelial diameter. However, the physiological properties of the cell membrane, including integrity, fluorescence intensity and permeability, were altered. A diagram was provided to demonstrate the behaviour of *Monascus* pigment secretion induced by Triton X‐100. This study lays a foundation for the further investigation of *Monascus* pigment metabolism and secretion in extractive fermentation.

## Introduction

*Monascus* fungi synthesize various functional pigments with polyketide structures that are widely utilized in the food and medicine industries (Patakova, 2013). *Monascus* pigments are a mixed group of azaphilones composed of yellow, orange and red compounds. Of these, two orange pigments (rubropunctatin and monascorubrin), two red pigments (rubropunctamine and monascorubramine) and two yellow pigments (monascin and ankaflavin) have been identified as hydrophobic pigments (Lin *et al*., 2008).

Pigment biosynthesis in *Monascus* is generally related to polyketide and fatty acid metabolism catalysed by polyketide synthase and fatty acid synthase (Feng *et al*., 2012). Recently, genomics and proteomics have been widely used to study the pigment synthesis and secretion pathways in depth. A putative gene cluster for the synthesis of these pigments has been reported (Balakrishnan *et al*., 2013). A homologue of genes and regulatory factors for transcription are also responsible for pigment metabolism (Patakova, 2013; Xie *et al*., 2013; Liu *et al*., 2014). However, detailed information of the pathways and enzymes involved in pigment biosynthesis remains unclear or controversial (Yang *et al*., 2015a). Furthermore, few studies about the process of pigment secretion are introduced, and a better understanding of the mechanism behind pigment secretion is also needed.

Similar to the solid‐state fermentation (Babitha *et al*., 2007a), pigment production through the submerged fermentation of *Monascus* fungi has also been studied intensively. Research reports in this area include the optimization of cultivation conditions (Babitha *et al*., 2007b; Orozco and Kilikian, 2008; Yang *et al*., 2015b) and medium composition (Subhasree *et al*., 2011; Hajjaj *et al*., 2012), as well as the development of novel fermentation techniques including high‐cell‐density fermentation (Chen *et al*., 2015), immobilized cell fermentation (Fenice *et al*., 2000) and extractive fermentation (Hu *et al*., 2012). Extractive fermentation with micelles of the non‐ionic surfactant Triton X‐100 in aqueous solution is an efficient method for *Monascus* pigments production. The intracellular hydrophobic pigments that are distributed in mycelia of conventional batch fermentation demonstrate transport behaviour through the cell membrane to extracellular non‐ionic surfactant micelles broth during extractive fermentation (Hu *et al*., 2012; Kang *et al*., 2013a). Moreover, it has been reported that the pH, Triton X‐100 concentration and timing of surfactant application play key roles in modulating the pigment characteristics during extractive fermentation (Kang *et al*., 2013b; Xiong *et al*., 2015). Although Triton X‐100 shows good biocompatibility with mycelia and can promote the extractability of pigments, its mechanism of influence on the cell membrane is unclear (Hu *et al*., 2012; Kang *et al*., 2013a).

To clarify the secretion process of *Monascus* pigments during extractive fermentation, an in‐depth investigation of their trans‐membrane behaviour was performed. The responses of mycelium morphology to the surrounding non‐ionic surfactant micelle aqueous solution and the variation of fungal membrane properties for pigment transport were studied accordingly.

## Results and Discussion

### Rate of pigment trans‐membrane behaviour induced by non‐ionic surfactant

**Figure 1:**
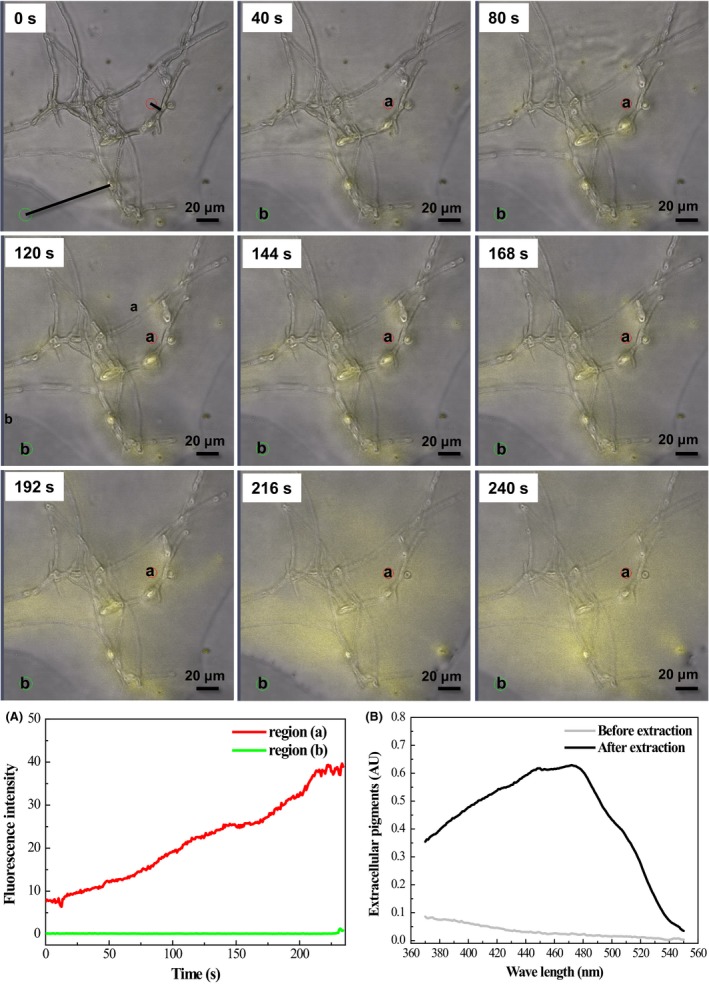
Trans‐membrane secretion of *Monascus* pigment in fermentation broth containing Triton X‐100. (A) Fluorescence intensity by region: (a) area near mycelium within 10 μm, (b) area distant from mycelium about 80 μm; (B) spectra of extracellular pigment before and after extraction.

When Triton X‐100 was added to the fermentation broth, the extracellular fluorescent substance responded rapidly, with the secretion of accumulated intracellular pigment through the cell wall (Fig. 1, 0‐240 s). The fluorescence intensity near the mycelium within 10 μm (region a) jumped instantaneously from 0 up to 8. After that, the fluorescence intensity increased linearly to a maximum value of approximately 40 in 240 s. At the areas 80 μm away from the mycelium (region b), the fluorescence intensity remained at approximately zero over the entire experiment (Fig. 1A). Thus, the fluorescence intensity in region (a) was increased by 2500% than that in region (b) after 240 s of extractive cultivation. This finding was well correlated with a 30‐fold greater secretion of pigment yields, when compared with the absorbance value at 470 nm before and after extraction (Fig. 1B). Most accumulated intracellular pigments were transferred to the outside of the cell rapidly, while only about 11% of pigments (0.081 AU_470_) remained in the mycelia. UV spectra (Fig. 1B) showed that pigments secreted into non‐ionic surfactant aqueous solution differed greatly from those collected from broth using traditional batch cultivation, but were quite similar to that of intracellular pigments, with a maximum absorption peak at approximately 470 nm (Chen *et al*., 2015). As the morphology of mycelia was maintained, the promoted secretion of intracellular pigments was likely caused by a change in cell membrane permeability due to the presence of Triton X‐100.

### Trans‐membrane conversion of the pigments of mature cells in a non‐ionic surfactant micelle aqueous solution

**Figure 2:**
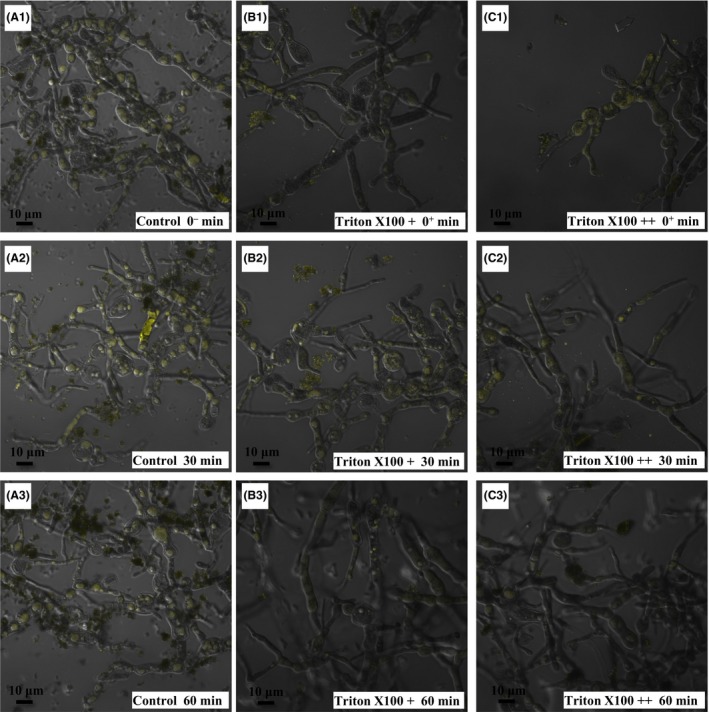
Fluorescence intensity of mature cells with varying concentrations of Triton X‐100 over time. A1, A2 and A3 represent mature cells in 0 g l^−1^ aqueous Triton X‐100 at 0, 30 and 60 min, respectively. B1, B2 and B3 represent mature cells in 15 g l^−1^ aqueous Triton X‐100 at 0, 30 and 60 min, respectively. C1, C2 and C3 represent mature cells in 40 g l^−1^ aqueous Triton X‐100 at 0, 30 and 60 min, respectively.

Relatively strong intracellular fluorescence was observed for mature mycelia (Fig. 2A1). This fluorescence showed good stability, as the intensity did not significantly (*P *>* *0.05) change over time (Table 1). An instantaneous decline in intracellular fluorescence occurred once mycelia were immersed in the non‐ionic surfactant Triton X‐100 aqueous solutions (Fig. 2B1 and C1), which was consistent with the image analysis mentioned above (Fig. 1). This decreasing tendency of fluorescence intensity induced by Triton X‐100 became more obvious after 60 min of extraction (Fig. 2B3 and C3). Notably, the higher the Triton X‐100 concentrations applied, the lower the fluorescence intensity observed (Table 1). This phenomenon illustrated a release of intracellular pigment attached to fluorescent substances from mycelia, and the amount of extracted pigment was correlated with the concentration of non‐ionic surfactant in the broth. These results are in agreement with those reported by Hu *et al*. (2012) that of the intracellular pigments extracted by Triton X‐100 were also concentration dependent. Moreover, both concentrations of Triton X‐100 led to a rapid decrease in fluorescence intensity with similar rates to reach equilibrium (Fig. 2B1‐B3, C1‐C3; Table 1). In our study of cellular morphology, there was no relative damage to the mycelium, with a completely intact mycelial shape in the Triton X‐100 non‐ionic surfactant solution similar to the aqueous control (Fig. 2). Although a significant (*P *<* *0.05) increase in mycelial diameter was observed with Triton X‐100, the change in mycelial diameter was not correlated with cultivation time (Table 1). Therefore, Triton X‐100 affects the permeability of the cell membrane, not only promoting the penetration of intracellular pigments to the outside broth but also resulting in the passage of extracellular water molecules into the mycelia.

**Table 1:** Variation of pigment fluorescence and mycelial diameter of mature cells in aqueous non‐ionic surfactant micelle solution

Triton X‐100 concentration (g l^−1^)	Extractive fermentation time (min)					
	Pigment fluorescence (%)			Mycelial diameter (μm)		
	0	30	60	0	30	60
0	5.01 ± 0.12Aa	5.46 ± 0.21Aa	5.62 ± 0.10Aa	3.78 ± 0.35Aa	3.66 ± 0.29Aa	3.73 ± 0.26Aa
15	3.51 ± 0.09Bb	3.66 ± 0.10Bb	2.57 ± 0.05Cc	5.06 ± 0.72Bb	4.99 ± 0.59Bb	5.00 ± 0.42Bb
40	2.66 ± 0.30Cc	3.20 ± 0.10Bb	2.33 ± 0.20Cc	4.71 ± 0.35 Bb	4.81 ± 0.39Bb	4.68 ± 0.32Bb

Data of pigment fluorescence are mean values ± standard deviations (*n* > 3). Data of mycelial diameter are mean values ± standard deviations (*n* > 30). Mean values with different lowercase letters (a, b, c) within a row are significantly different (*P *<* *0.05); mean values with different capital letters (A, B, C) within a column are significantly different (*P *<* *0.05).

**Figure 3:**
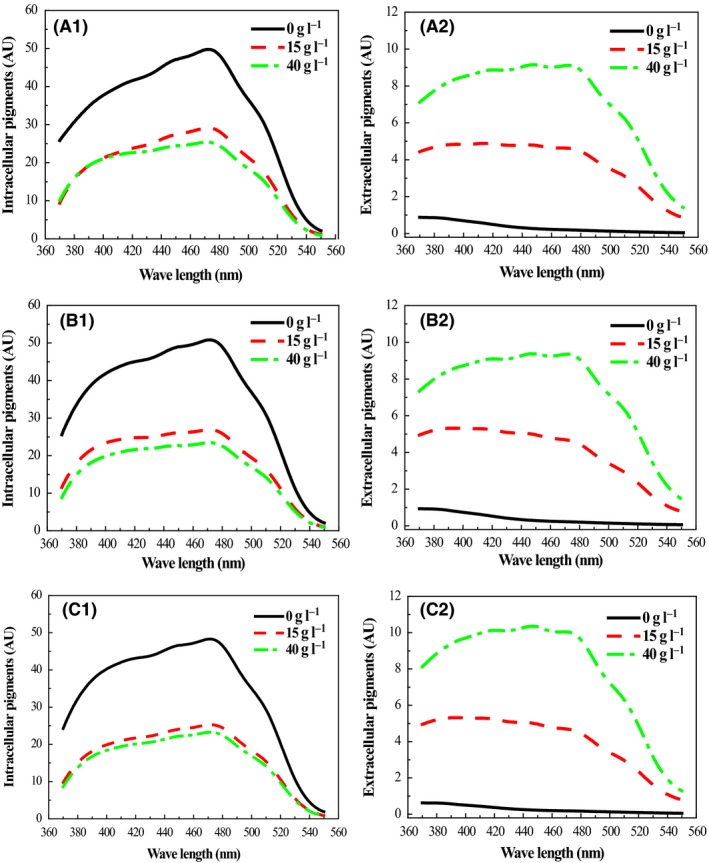
Characteristic variation in intracellular and extracellular pigments extracted using various concentrations of Triton X‐100 over time. A1, B1 and C1 represent intracellular pigments extracted by 0, 15 and 40 g l^−1^ aqueous Triton X‐100 at 0, 30 and 60 min, respectively. A2, B2 and C2 represent extracellular pigment extracted with 0, 15 and 40 g l^−1^ aqueous Triton X‐100 at 0, 30 and 60 min, respectively.

The distribution patterns of intracellular and extracellular pigment in the mycelium and broth were studied. The AU_470_ of intracellular pigments in mature cells was approximately 50 in aqueous solution, but instantaneously dropped to approximately 30 in 15 g l^−1^ non‐ionic surfactant Triton X‐100 aqueous solution and further declined to 25 with a higher surfactant concentration of 40 g l^−1^ (Fig. 3A1). In comparison, the absorbance of extracellular pigments had a very low value of 0.6 AU_410_ in aqueous solution (0 g l^−1^ Triton X‐100), and its spectrum showed a unilateral decline curve from 370 to 550 nm (Fig. 3 A2). Absorbance simultaneously increased to approximately 5 AU_410_ in 15 g l^−1^ Triton X‐100 aqueous solutions. With the increased concentration of Triton X‐100 to 40 g l^−1^, the extracellular pigment monotonically increased to 9 AU_410_ (Fig. 3 A2). The increase in the amount of extracellular pigment as intracellular pigment decreased indicated that intracellular pigments travelled across the mycelial membrane to the broth. Using Triton X‐100, the UV patterns of extracellular and intracellular pigment over three extraction times (0, 30 and 60 min) were very similar (Fig. 3), revealing that the extraction of intracellular pigment occurs rapidly. Interestingly, it was not possible to completely extract all intracellular pigment from the mycelium, even with higher concentrations of Triton X‐100 and longer extraction times (Fig. 3C1). This limit of extractive saturation in non‐ionic surfactant micelle aqueous solutions is supported by our early work, wherein the yield of extracellular pigments ranged from 40% to 60% of the total pigments using 40 g l^−1^ Triton X‐100 (Chen *et al*., 2017a). The spectral patterns of intracellular pigments in aqueous solution were similar to those in Triton X‐100 solution, with an absorbance maximum at 470 nm (Fig. 3, A1‐C1), the characteristic adsorption of orange pigment (Zheng *et al*., 2009). In contrast, the absorbance maximum of the extracted extracellular pigment gradually shifted from 390 to 470 nm (Fig. 3, A2‐C2), distinct from the extracellular pigments in the traditional batch cultivation (Chen *et al*., 2015). Therefore, the pigment characteristics were altered by the membrane transport process. Moreover, the increase in extracellular orange pigment was not equal to the decrease in intracellular orange pigment (Fig. 3). A conversion between orange and yellow pigment (Chen *et al*., 2017b) is proposed to explain the differential efficiency of rapid transfer through the cell membrane.

### Altered cell membrane and mycelium structure in non‐ionic surfactant micelle aqueous solution

**Figure 4:**
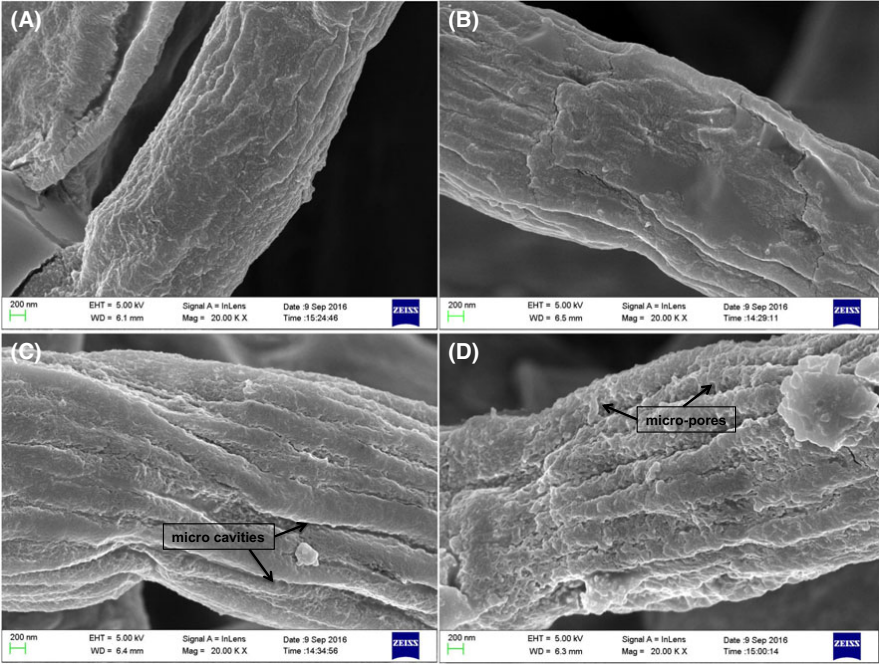
SEM image of mycelium morphology at different concentrations of Triton X‐100 aqueous solution. (A) 0 g l^−1^, (B) 15 g l^−1^, (C) 40 g l^−1^, (D) 80 g l^−1^.

Scanning electron microscope (SEM) images showed a smooth cell wall surface with no significant damage in the batch culture (Fig. 4A). An uneven surface profile with small fragments appeared at a low concentration (15 g l^−1^) of Triton X‐100 (Fig. 4B). At 40 g l^−1^ Triton X‐100, the cell wall exhibited more cracks, and the fibrous tissue was partially dissolved, with evidence of micro‐cavities (Fig. 4C). The fibrous tissue took on an irregular shape, and a large number of micro‐pores were found distributed on the surface of the mycelium at 80 g l^−1^ Triton X‐100 (Fig. 4D). This indicates that the non‐ionic surfactant Triton X‐100 can modify the cell walls of *Monascus* mycelium and increase cell membrane permeability to facilitate the secretion of intracellular pigments (Kang *et al*., 2013b).

**Figure 5:**
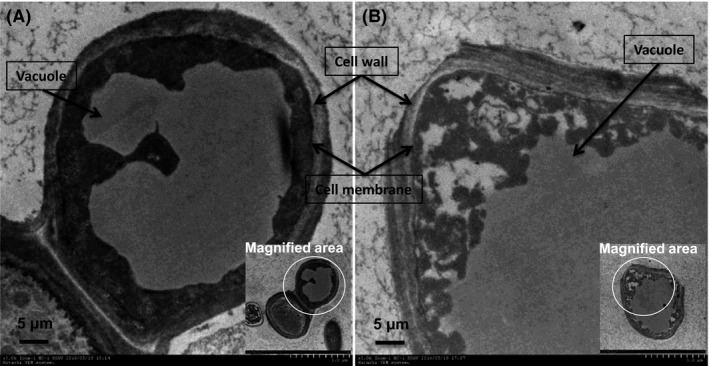
TEM image of cell internal structure at different concentrations of Triton X‐100 aqueous solution. (A) 0 g l^−1^, (B) 40 g l^−1^, the magnified viewing area is pointed out by the white circle.

Transmission electron microscopy (TEM) was used to investigate the change in mycelial cell membranes and internal structure. In control fermentations without Triton X‐100, the contours of the cell wall and membrane were relatively clear. Vacuoles that act as repositories for intracellular pigments (Glover and Martin, 1998; Suh and Shin, 2000) were also undamaged (Fig. 5A). Although the cell membrane remained intact at 40 g l^−1^ Triton X‐100, the structure of the cell wall and vacuole was destroyed, and the internal contents, including pigments, were irregularly distributed and released into the cytosol in the mycelium (Fig. 5B). These changes in cytoarchitectural features indicate a penetration of Triton X‐100 into the mycelium that enhances the release of intracellular pigment from repositories.

### Release of intracellular pigment via the leakage of cytoplasm

**Figure 6:**
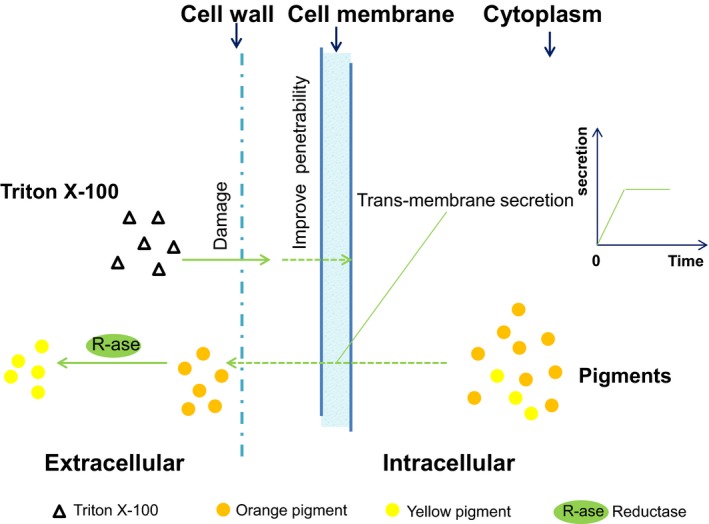
Putative diagram of the trans‐membrane secretion of *Monascus* pigment in extractive fermentation.

Organic matter such as Tween‐80, toluene, ether and chitosan can increase cell membrane penetrability and cause the leakage of cytoplasm (Chen *et al*., 2007; Tao *et al*., 2011). Thus, we investigated the cytoplasm leakage of *Monascus* in Triton X‐100 micelle aqueous solution. The specific absorbance values at 260 and 280 nm for nucleic acids increased by approximately 19% and 17%, respectively (Table 2). The increased nucleic acid content indicates damage leading to the leakage of cytoplasm. Moreover, the electrical conductivity of the extractive solution was fourfold higher than that in the control fermentation broth (Table 2), indicating a leakage of ions from the mycelia. Multiple ion channels for Na^+^, K^+^, Ca^+^ and Cl^−^ exist in the cell membrane (Al‐Rawahy and Farooq, 2012) and not only regulate osmotic balance but also maintain the membrane potential (Zha *et al*., 2014; Silva and Silverira, 2015). Cell membrane ion channels are sensitive to Triton X‐100, which may lead to the disruption of the normal transfer of K^+^ and Cl^−^. The leakage of other intracellular matter including pigments, proteins, amino acids and other ions may also alter electrical conductivity. Furthermore, it is reported that the tryptophan (Trp) and tyrosine (Tyr) residues in membrane proteins are fluorescent (Gleason *et al*., 2013). Variations in this fluorescence directly reflect either changes in their concentration or a conformational change in the membrane proteins (Nicholas and Anthis, 2013). In this study, the fluorescence intensity of Trp and Tyr was slightly increased by approximately 7% and 5%, respectively, when Triton X‐100 was added to the fermentation broth (Table 2). Triton X‐100 may promote a conformational change for membrane proteins that exposes tryptophan and tyrosine residues on the extracellular side of cell membrane, disrupting the protein structure facing the aqueous solution. Both Triton X‐100 and *Monascus* pigments are likely to be taken up and excreted mainly through trans‐membrane transport, as most small molecules accumulated in the vacuole are released through this route (Kleinegris *et al*., 2011). Thus, Triton X‐100 may affect cell membrane permeability by changing both the ion channel and the conformation of cell membrane proteins to facilitate the trans‐membrane transport of intracellular pigments. Our previous work had found a reduction reaction was involved in pigment conversion from orange pigments to yellow pigments that occurred in non‐aqueous phase solution (Chen *et al*., 2017b,c). On the basis of the facts mentioned above, we established a putative diagram of pigment trans‐membrane secretion in extractive fermentation (Fig. 6). However, this proposed mechanism for the enhanced extraction of pigment due to Triton X‐100 also requires further investigation.

**Table 2:** Variation of nucleic acid, Trp and Tyr fluorescence intensity and electrical conductivity with the addition of Triton X‐100

Cultivation mode	Nucleic acid		Fluorescence intensity		Electrical conductivity
	OD_260_	OD_280_	Trp	Tyr	
Before extraction (control)	1	1	1	1	1
After extraction	1.19 ± 0.02	1.17 ± 0.01	1.07 ± 0.01	1.05 ± 0.01	4.21 ± 0.05

Data are mean values ± standard deviations (*n* = 3).

## Conclusions

Pigment secretion in extractive fermentation significantly differed from that in traditional batch fermentation. Coupled with damage in cell walls, the penetrability of the cell membrane was increased, and then, the intracellular pigment rapidly extracted into the extracellular pigment in aqueous non‐ionic surfactant Triton X‐100 micelles solution. In trans‐membrane process, the intracellular pigments were secreted with characteristic conversion. The extraction capacity of pigments was limited by the concentration of the non‐ionic surfactant applied. Therefore, a putative diagram was provided to demonstrate the behaviour of trans‐membrane secretion of *Monascus* pigment in extractive fermentation.

## Experimental procedures

### Microorganism and traditional batch culture

*Monascus anka* GIM 3.592 that deposited in the publicly accessible culture collection Guangdong Culture Collection Centre of Microbiology (GDMCC/GIMCC, China) was maintained on potato dextrose agar medium (potato dextrose 200 g, glucose 20 g and agar 15–20 g per litre of distilled water) and preserved at 4 °C. Each month, a subculture was carried out at 30 °C for 7 days.

The inoculum culture medium consisted of glucose (20 g), yeast extract (3 g), peptone (10 g), KH_2_PO_4_ (4 g), KCl (0.5 g) and FeSO_4_·7H_2_O (0.01 g) per litre of distilled water. The inoculum culture was prepared in a 250 ml Erlenmeyer flask containing 50 ml of the seed culture medium and incubated at 30 °C for 30 h in a rotary shaker with 180 rpm.

The fermentation culture medium consisted of glucose (50 g), (NH_4_)_2_SO_4_ (5 g), KH_2_PO_4_ (5 g), MgSO_4_·7H_2_O (0.5 g), KCl (0.5 g), MnSO_4_·H_2_O (0.03 g), ZnSO_4_·7H_2_O (0.01 g) and FeSO_4_·7H_2_O (0.01 g) per litre of distilled water. The initial pH of the fermentation medium was natural. The inoculum culture (2 ml) was inoculated into 25 ml of fermentation medium in 250 ml Erlenmeyer flasks for submerged fermentation. The batch culture was cultivated at 30 °C and 180 rpm for 6 days.

The potato dextrose and yeast extract were purchased from the Aoboxing Bio‐Tech (Beijing, China) and Oxoid (Hants, UK), respectively. The agar and peptone were purchased from the Sinopharm Chemical Reagent (Shanghai, China). All other chemicals and reagents were of analytical grade and also purchased from the Sinopharm Chemical Reagent.

### Secretion of pigments in non‐ionic surfactant aqueous solution

Tests to quantitate pigment secretion were performed as follows: fermentation broth that was cultivated for 6 days was removed and diluted 100‐fold with distilled water to an absorbance at 600 nm approximately 0.2–0.25. Next, 300 μl of diluent was placed in a petri dish to observe mycelium morphology, pigment distribution and fluorescence intensity using LSCM (Zeiss LSM 710, Jena, Germany) with an excitation wavelength of 488 nm and an emission wavelength of 542–573 nm. Next, 100 μl of 60 g l^−1^ aqueous Triton X‐100 was added to the petri dish to observe the secretion process of fluorescence pigments from mycelia to extracellular broth in real time. Samples of test broth before and after the addition of Triton X‐100 were collected and centrifuged at 10 000 rpm for 5 min, respectively. The supernatant was diluted to determine the extracellular pigment concentration, and the mycelia were then soaked in a 70% (V/V) ethanol aqueous solution (adjustment of pH to 2 with hydrochloric acid) for 1 h to extract and determine the residual intracellular pigment concentration.

### Extractive cultivation of mature cells for extracellular pigments

First, 5 ml of fermentation broth cultivated for 6 days was removed and centrifuged at 10 000 rpm for 5 min. The collected mycelia were divided into three groups, soaked in 0, 15 and 40 g l^−1^ aqueous Triton X‐100 in a total volume of 5 ml and incubated at 30 °C at 180 rpm for 0, 30 and 60 min, respectively. The extractive cultures were centrifuged at 10 000 rpm for 5 min to separate the mycelia, and the supernatants were diluted to determine the extracellular pigment concentration. The mycelia were detected using LSCM to observe the pigment fluorescent and mycelium diameter, and then were collected to determine the residual intracellular pigment concentration mentioned above.

The pigment fluorescence observed by LSCM was performed at least three times and expressed as relative values, which defined as the percentage of the fluorescence area to the entire field of mycelia. In every repeated experiment, the mean diameter of about 10 randomly selected mycelia was measured using the image analysis software ZNE 2012 SP2 (Carl Zeiss, Jena, Germany).

### Mycelium morphological analysis after extractive cultivation

#### Scanning electron microscopy (SEM)

First, 1 ml of fermentation broth cultivated for 6 days was removed and centrifuged at 8000 rpm for 5 min. The mycelia were collected, washed three times with distilled water and soaked in 0, 15, 40 and 80 g l^−1^ aqueous Triton X‐100 for 1 h. The mycelia were separated by centrifugation at 8000 rpm for 5 min and washed three times with 0.1 M PBS buffer. The mycelia were fixed with 1 ml of 2.5% glutaraldehyde (dissolved in 0.1 M PBS buffer) for 4 h. The mycelia were collected by centrifugation at 8000 rpm for 5 min and washed three times with 0.1 M PBS buffer to remove residual glutaraldehyde. The mycelia were re‐suspended with 1 ml of 0.1 M PBS buffer, freeze‐dried overnight and imaged using SEM (Merlin; Zeiss).

#### Transmission electron microscope (TEM)

Mycelia were prepared similar to the SEM method, but the concentrations of aqueous Triton X‐100 were 0 and 40 g l^−1^. The mycelia were fixed with 1 ml of fixative (4% glutaraldehyde and 3% paraformaldehyde) for 4 h. The mycelia were collected by centrifugation at 8000 rpm for 5 min and washed three times with 0.1 M PBS buffer to remove residual fixative. The mycelia were fixed with 0.1 ml of 1% osmic acid overnight, washed four times with 0.1 M PBS buffer and dehydrated successively with 30%, 50%, 70%, 85%, 95% and 100% (v/v) ethanol. The mycelia were embedded in resin and further polymerized at 65 °C for 2 days. The treated samples were sliced using an ultra‐microtome, stained with uranium acetate and lead citrate, and observed using TEM (H‐600; Hitachi, Tokyo, Japan).

### Investigation of cell membrane physiological properties after extractive fermentation

#### Fluorescence intensity assay of cell membranes

Fermentation broth cultivated for 6 days was removed and diluted 100‐fold with distilled water to adjust the absorbance to 0.2–0.25 at 600 nm. Next, 1.5 ml of diluent was mixed with 0.5 ml of 60 g l^−1^ Triton X‐100 or distilled water (control). The mixture was centrifuged at 10 000 rpm for 5 min to clarify the supernatant for fluorescence intensity analysis. Absorbance was detected using a fluorescence spectrophotometer (Spectra Max M5, Molecular Devices, California, USA) with an excitation wavelength of 297 or 292 nm, corresponding to Trp or Tyr, respectively, and an emission wavelength of 348 nm (Ye *et al*., 2007).

#### Cell membrane permeability analysis

Sample preparation was as above, and 1.5 ml of distilled water was also mixed with 0.5 ml of 60 g l^−1^ Triton X‐100 as a control. The supernatant was removed (1 ml) and mixed with 4 ml of distilled water to measure electrical conductivity. The electrical conductivity was detected using an electric conductivity meter (DDS‐307A, Shanghai Precision Science Instrument, Shanghai, China) to characterize cell membrane permeability (Ye *et al*., 2005).

#### Cell membrane integrity determination

Sample preparation was as above, but the 0.5 ml of distilled water was replaced by 1% aqueous acetic acid. The supernatant was removed to directly detect the nucleic acid. Nucleic acid concentration, which is a proxy for cell membrane integrity, was detected using an ultraviolet spectrophotometer (UV‐2802S, UNICO, Shanghai, China) at 260 and 280 nm (Xing *et al*., 2009).

All the results of fluorescence intensity, permeability and integrity analysis of cell membranes were expressed as relative values between before and after extraction, and the former (before extraction) was considered the control as the reference value (value 1).

#### Pigments concentration analysis

The estimation of pigment concentration in this study followed the method detailed in our previous work (Chen *et al*., 2015). *Monascus* pigments are a group of mixed colours containing different compositions, which is difficult to quantify the concentration in grams or moles (Lin *et al*., 2008; Feng *et al*., 2012; Patakova, 2013). Generally, the contents of *Monascus* pigments were evaluated by their integrated colour characteristics through the visible absorbance at 410, 470 and 510 nm for yellow, orange and red pigments, respectively (Hajjaj *et al*., 2012; Kang *et al*., 2013a,b). The results were represented in absorbance units (AU, multiplication of absorbance by dilution ratio for a given sample).

### Statistical analysis

Data were expressed as the mean values ± standard deviation (SD) for each measurement. The data were submitted to anova analysis, and significance of differences was determined by Duncan's multiple range tests where necessary. *P *<* *0.05 was considered statistically significant in all cases. All the analysis was carried out with the spss software package (version 22.0; SPSS, Chicago, IL, USA).

## Conflict of interest

None declared.
